# Platelet Protein-Related Abnormalities in Response to Acute Hypoglycemia in Type 2 Diabetes

**DOI:** 10.3389/fendo.2021.651009

**Published:** 2021-03-30

**Authors:** Abu Saleh Md Moin, Ahmed Al-Qaissi, Thozhukat Sathyapalan, Stephen L. Atkin, Alexandra E. Butler

**Affiliations:** ^1^ Diabetes Research Center (DRC), Qatar Biomedical Research Institute (QBRI), Hamad Bin Khalifa University (HBKU), Qatar Foundation (QF), Doha, Qatar; ^2^ Academic Endocrinology, Diabetes and Metabolism, Hull York Medical School, Hull, United Kingdom; ^3^ Academic Endocrinology, Leeds Medical School, Leeds, United Kingdom; ^4^ School of Postgraduate Studies and Research, Royal College of Surgeons in Ireland Bahrain, Adliya, Bahrain

**Keywords:** type 2 diabetes, obesity, platelet, COVID-19, SARS-CoV-2

## Abstract

**Introduction:**

Patients with severe COVID-19 infections have coagulation abnormalities indicative of a hypercoagulable state, with thromboembolic complications and increased mortality. Platelets are recognized as mediators of inflammation, releasing proinflammatory and prothrombotic factors, and are hyperactivated in COVID-19 infected patients. Activated platelets have also been reported in type 2 diabetes (T2D) patients, putting these patients at higher risk for thromboembolic complications of COVID-19 infection.

**Methods:**

A case-control study of T2D (n=33) and control subjects (n=30) who underwent a hyperinsulinemic clamp to induce normoglycemia in T2D subjects: T2D: baseline glucose 7.5 ± 0.3mmol/l (135.1 ± 5.4mg/dl), reduced to 4.5 ± 0.07mmol/l (81 ± 1.2mg/dl) with 1-hour clamp; Controls: maintained at 5.1 ± 0.1mmol/l (91.9 ± 1.8mg/dl). Slow Off-rate Modified Aptamer (SOMA)-scan plasma protein measurement was used to determine a panel of platelet proteins.

**Results:**

Prothrombotic platelet proteins were elevated in T2D versus controls: platelet factor 4 (PF4, p<0.05); platelet glycoprotein VI (PGVI p<0.05); P-selectin (p<0.01) and plasminogen activator inhibitor I (PAI-1, p<0.01). In addition, the antithrombotic platelet-related proteins, plasmin (p<0.05) and heparin cofactor II (HCFII, p<0.05), were increased in T2D. Normalization of glucose in the T2D cohort had no effect on platelet protein levels.

**Conclusion:**

T2D patients have platelet hyperactivation, placing them at higher risk for thromboembolic events. When infected with COVID-19, this risk may be compounded, and their propensity for a more severe COVID-19 disease course increased.

**Clinical Trial Registration:**

https://clinicaltrials.gov/ct2/show/NCT03102801, identifier NCT03102801.

## Introduction

Patients with severe COVID-19 infections have coagulation abnormalities indicative of a hypercoagulable state, with thromboembolic complications and increased mortality (1). What underlies this hypercoagulable state has not been fully elucidated, although it is likely due to a combination of endothelial injury (direct invasion by SARS-CoV-2), the inflammatory response mediated largely by cytokines, patient immobility and altered circulating clotting proteins (decreased antithrombin combined with increased prothrombotic proteins like D-dimer, factor VIII, fibrinogen and fibrin degeneration products) (1).

Platelets are essential for hemostasis, but they are also involved in detrimental sequelae such as myocardial infarction, stroke and deep vein thrombosis (2–5). Type 2 diabetes (T2D) patients have activated platelets that contribute to the increased cardiovascular disease in T2D (6, 7). Platelets are known mediators of inflammation, and release proinflammatory and prothrombotic molecules such as CD40 ligand and thromboxane A2 (8). Oxidative stress markers are generated in the euglycemic state (9), but it is recognized that glucose variability and in particular hyperglycemia may increase inflammatory markers and oxidative stress through effects on the polyol and hexosamine pathways and increased protein kinase C activation *via* diacyglycerol (10–12).

Thrombocytopenia has emerged as a complication of COVID-19 infection; this, together with increased D-dimer levels, is likely due to hyperactivation of platelets and the coagulation cascade (1, 13).

We hypothesized that circulating levels of platelet-related proteins would be altered in T2D, priming these patients for an upregulated thromboembolic response to infection, and that euglycemia would lead to normalization of these platelet-related proteins. We therefore determined levels of a panel of platelet-related proteins in plasma in T2D compared to control subjects, and subsequently compared these proteins in T2D at baseline to those when euglycemia was re-established.

## Methods

A case-control study of T2D (n=33) and control subjects (n=30), approved by Yorkshire and Humber Research Ethics Committee, was performed. The hyperinsulinemic clamp was performed as previously described (14). Subjects were matched for age (p=ns) though BMI was higher in T2D (p<0.0001). For T2D patient inclusion, only metformin as anti-diabetic therapy was allowed.

The duration of diabetes in the T2D cohort was 4.2 ± 0.4 years. All participants were Caucasian who fasted 10-hours before venipuncture. In the T2D cohort, the baseline glucose of 7.5 ± 0.3mmol/l (135.1 ± 5.4mg/dl) was reduced to 4.5 ± 0.07mmol/l (81 ± 1.2mg/dl) with the 1-hour clamp whilst the control subjects were maintained at 5.1 ± 0.1mmol/l (91.9 ± 1.8mg/dl).

Slow Off-rate Modified Aptamer (SOMA)-scan plasma protein measurement (15) was used to determine platelet-related proteins: platelet factor 4 (PF4), platelet glycoprotein VI (PGVI), P-selectin, plasminogen activator inhibitor I (PAI-1), plasmin and heparin cofactor II protein concentrations, expressed as relative fluorescent units (RFU).

Statistics were performed with GraphPad Prism 8.0.

## Results

As anticipated, HbA1c was elevated in T2D (51 ± 2 vs 37 ± 0.5 mmol/mol [6.8 ± 2 vs 5.5 ± 2%], p<0.0001) Platelet count and C-reactive protein did not differ between T2D and control subjects (p=ns) (**Table 1**).

**Table 1:** Demographic and clinical characteristics of the study participants.

Baseline	Type 2 Diabetes (n = 23)	Controls (n = 23)	p-value
Age (years)	64 ± 8	60 ± 10	<0.0001
Sex (M/F)	12/11	11/12	0.77
Weight (kg)	90.9 ± 11.1	79.5 ± 8.8	<0.0001
Height (cm)	167 ± 14	169 ± 5	0.64
BMI (kg/m^2^)	32 ± 4	28 ± 3	<0.0001
Systolic BP (mmHg)	132 ± 8	122 ± 8	0.001
Diastolic BP (mmHg)	81 ± 7	75 ± 6	0.003
Duration of diabetes (years)	4.5 ± 2.2	N/A	
HbA1c (mmol/mol)	51.2 ± 11.4	37.2 ± 2.2	<0.0001
HbA1c (%)	6.8 ± 1.0	5.6 ± 0.2	<0.0001
Total cholesterol (mmol/l)	4.2 ± 1.01.0	4.8 ± 0.77	0.014
Triglyceride (mmol/l)	1.7 ± 0.7	1.34 ± 0.6	0.055
HDL-cholesterol (mmol/l)	1.1 ± 0.3	1.5 ± 0.4	0.001
LDL-cholesterol (mmol/l)	2.23 ± 0.8	2.7 ± 0.87	0.051
CRP (mg/l)	3.10 ± 2.87	5.30 ± 1110.03	0.66
Platelet count (x10^9^/l)	270 ± 77	240 ± 48	0.07

BMI, Body mass index; BP, Blood pressure; HDL-cholesterol, High density lipoprotein cholesterol; LDL-cholesterol, Low density lipoprotein cholesterol; CRP, C-reactive protein. HbA1c, Hemoglobin A1c.

The following prothrombotic platelet-related proteins were increased in T2D versus controls: PF4 (76548 ± 15982 vs 37645 ± 5704 RFU, p<0.05); PGVI (4251 ± 331 vs 3567 ± 192 RFU, p<0.05); P-selectin (14030 ± 773 vs 11255 ± 467 RFU, p<0.01); PAI-1 (2161 ± 225 vs 1341 ± 208 RFU, p<0.01).

**Figure 1:**
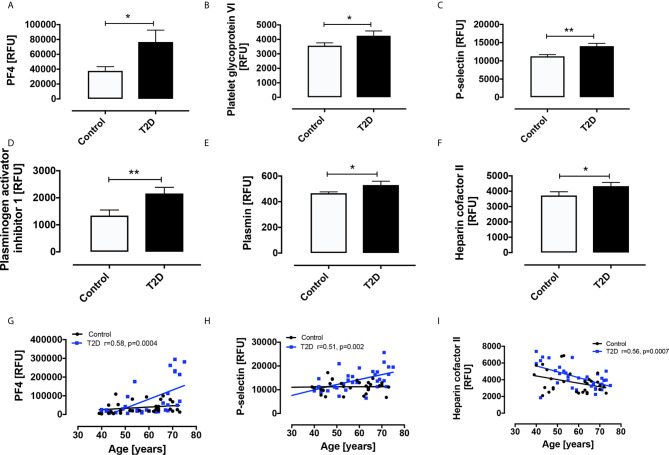
Circulatory levels of platelet related proteins in obese type 2 diabetes (OT2D) and control subjects. Plasma protein levels of platelet factor 4 (PF4) **(A)**, platelet glycoprotein VI (PGVI) **(B)**, P-selectin **(C)**, plasminogen activator inhibitor I (PAI-1) **(D)**, plasmin **(E)**, plasmin and heparin cofactor II **(F)**. Age correlated with levels of PF4 (r = 0.58) **(G)**, P-selectin (r = 0.51) **(H)** and Heparin cofactor II (r = 0.56) **(I)** in the T2D cohort only. RFU: relative fluorescent units; *p < 0.05; **p < 0.01.

By contrast, the antithrombotic platelet-related proteins increased in T2D versus controls were plasmin (530 ± 30 vs 466 ± 11 RFU, p<0.05) and heparin cofactor II (4331 ± 241 vs 3720 ± 242 RFU, p<0.05) (**Figures 1A–F**).

Contrary to our hypothesis, normalization of glucose in the T2D cohort had no effect on levels of any of the plasma platelet-related proteins.

Notably, age correlated with levels of PF4 (r=0.58, p=0.0004), P-selectin (r=0.51, p=0.002) and Heparin cofactor II (r=0.56, p=0.0007) in the T2D cohort only (**Figures 1G–I**).

**Figure 2:**
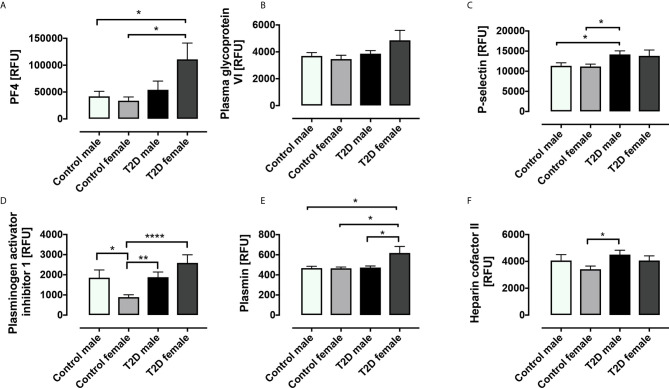
Circulatory levels of platelet related proteins in obese type 2 diabetes (OT2D) and control subjects stratified according to gender. Plasma protein levels of platelet factor 4 (PF4) **(A)**, platelet glycopsrotein VI (PGVI) **(B)**, P-selectin **(C)**, plasminogen activator inhibitor I (PAI-1) **(D)**, plasmin **(E)**, plasmin and heparin cofactor II **(F)**. RFU: relative fluorescent units; *p < 0.05; **p < 0.01; ****p < 0.0001.

Stratification of the T2D and control subjects into gender subgroups revealed some interesting gender-related differences in platelet-related protein levels (**Figure 2**). The T2D group was composed of 20 males and 13 females; in the control group, there were 14 males and 16 females. PF4 was increased in T2D females versus control females (p=0.012) and versus control males (p=0.035); there was a trend versus T2D males, but this did not reach significance (p=0.084). PGVI showed a trend towards increase in T2D females versus control females (p=0.071) though this did not reach significance. P-selectin was significantly elevated in T2D male versus control male (p=0.028) and versus control female (p=0.012) subjects; again, T2D females showed a trend towards increase versus control females, though this did not reach significance (p=0.085). PAI-1 was significantly elevated in both T2D females and T2D males versus control females (p=0.0001 and p=0.003, respectively); PAI-1 was also significantly elevated in control males versus control females (p=0.018). Plasmin was elevated in T2D females versus control females (p=0.018), control males (p=0.030) and T2D males (p=0.014). Heparin cofactor II was increased in T2D males versus control females (p=0.014).

## Discussion

This study shows that the levels of multiple platelet-related proteins are increased in T2D.

PF4 and P-selectin are released from activated platelets (16), PGVI is a platelet procoagulant mediator (17) and elevated PAI-1 is a thrombosis risk factor (18). By contrast, plasmin degrades fibrin clots (19) and heparin cofactor II inhibits thrombin (20).

Taken together, these results indicate that T2D patients have an elevated platelet activation baseline and, therefore, any additional insult such as infection would accelerate and enhance their hypercoagulable state.

COVID-19-infected individuals with acute respiratory distress syndrome (ARDS) are also in a hypercoagulable state, evidenced by increased fibrinogen and fibrin degradation products (D-dimers); this hypercoagulability may underlie organ failure and death (21).

The results of the hyperinsulinemic clamp showed that this enhanced platelet activation seen was not improved by returning to euglycemia in the T2D subjects. It is possible that the one-hour insulin clamp was insufficient to alter some of the proteins associated with platelet function to euglycemia and a longer time course may have resulted in changes, perhaps elicited through inflammatory or oxidative stress mechanisms due to the change in glucose levels (22).

Interesting gender differences were also revealed by the gender-stratified subgroup analysis. In the case of PF-4, PAI-1 and plasmin, the elevation in these proteins in the T2D females drove the difference between T2D and controls in the whole group analysis; likewise, there was a trend for increase in T2D females for PGVI, though this did not reach significance. Conversely, for P-selectin and heparin cofactor II, it was the T2D males who predominantly drove the differences seen between T2D and controls in the whole group analysis. This is in keeping with other studies where proteomic analysis has shown a multitude of gender-related differences in platelet proteins (23–25), even suggesting mechanistic links to diseases with known gender disparities, such as the increased prevalence of cardiovascular disease in males (24).

Study strengths are that the T2D subjects had been diabetic for only a relatively short duration. Study limitations include small study numbers and, with more subjects enrolled, greater differences in platelet-related proteins between T2D and control subjects may have been apparent. Although T2D subjects were older and with higher BMI, this would not likely have altered protein levels. The lack of any changes from baseline in platelet-related proteins in response to normalization of glucose levels in the T2D subjects could perhaps be a consequence of the length of this clamp study being insufficient to reveal shifts in protein levels; therefore, future studies should be designed to maintain euglycemia for a longer period to better address this question. Furthermore, to conclusively determine the changes in platelet proteins in both T2D and controls in response to COVID-19 disease, it is necessary to analyze plasma from subjects documented to have SARS-CoV-2 infections and, ideally, to assess currently infected patients who have previously participated in clinical trials.

In conclusion, T2D patients have platelet hyperactivation, placing them at higher risk for thromboembolic events. When infected with COVID-19, this risk may be compounded, and their propensity for a more severe COVID-19 disease course increased.

## Data Availability Statement

The raw data supporting the conclusions of this article will be made available by the authors, without undue reservation.

## Ethics Statement

The studies involving human participants were reviewed and approved by The Yorkshire and Humber Research Ethics Committee. The patients/participants provided their written informed consent to participate in this study.

## Author Contributions

AM and AB analyzed the data and wrote the manuscript. AA-Q performed the clinical studies. TS supervised clinical studies and edited the manuscript. SA contributed to study design, data interpretation and the writing of the manuscript. AB is the guarantor of this work. All authors contributed to the article and approved the submitted version.

## Conflict of Interest

The authors declare that the research was conducted in the absence of any commercial or financial relationships that could be construed as a potential conflict of interest.
